# Hail netting: an economically competitive IPM alternative to insecticides for Midwest apple production

**DOI:** 10.3389/finsc.2023.1266426

**Published:** 2023-10-26

**Authors:** Gigi DiGiacomo, Sally G. A. Nelson, John Jacobson, Annie Klodd, William D. Hutchison

**Affiliations:** ^1^ Dept. of Applied Economics, University of Minnesota, St. Paul, MN, United States; ^2^ Dept. of Entomology, University of Minnesota, St. Paul, MN, United States; ^3^ Pine Tree Apple Orchard, White Bear Lake, MN, United States; ^4^ Minnesota Landscape Arboretum, University of Minnesota, Chaska, MN, United States

**Keywords:** hail netting, integrated pest management (IPM), apples, partial budgeting, net revenue, insecticide alternatives, exclusion

## Abstract

Apple orchards are highly managed agricultural ecosystems where growers typically rely on insecticides to minimize the risk of pest-related fruit losses. Apple growers practicing integrated pest management require cost-effective alternatives to conventional insecticides for control of major pests such as codling moth (*Cydia pomonella* L.) and apple maggot (*Rhagoletis pomonella* Walsh). Exclusion netting has been shown to effectively control multiple insect pest species, limit fruit damage and reduce the use of insecticides while also conferring consumer and environmental benefits. In this study, partial budgeting was applied to explore the financial efficacy of using a hail netting (DrapeNet^®^) system as a sustainable pest management strategy for Midwest U.S. apple (*Malus x domestica*). The cost of the hail netting was compared to a common Midwest insecticide spray regimen for apples using yield and quality data from a field study at two Minnesota apple orchards in 2021-2022. The PB analysis indicated that the netting system was an economically competitive alternative to conventional insecticide applications. The economic results were robust across a range of apple prices and yields suggesting that Minnesota apple growers can benefit economically from the application of hail netting for sustainable pest management.

## 1 Introduction

Sound pest management strategies are needed for Midwest U.S. apple (*Malus x domestica*) orchards due to substantial insect pest pressure. Most growers continue to rely on conventional pesticides for managing the most common major insect pests in apple orchards such as the codling moth (*Cydia pomonella* L.) and apple maggot (*Rhagoletis pomonella* Walsh), as well as less abundant secondary pest species (1). Following a conventional calendar-based spray schedule, costly insecticide applications for apple maggot have historically been applied every 10-14 days, averaging up to 10 applications per year globally (2–5). In Minnesota, conventional apple growers report applying insecticides more than 7 times on average throughout the production season (1).

The increasing cost of annual insecticide spray regimens and the potential for the evolution of insecticide resistance has Midwest fruit growers calling for more organic and non-chemical pest management strategies (6). The need for these strategies is becoming ever more urgent as climate change data reveal warming winter temperatures in Minnesota (7) that allow key apple pests such as codling moth to thrive (8). Increased pest pressure, without effective biological control or pesticide alternatives, can facilitate the evolution of resistance to various classes of insecticides and biologicals (9–11). Research indicates that simply scaling up conventional fruit production systems that rely on the use of insecticides, will hinder the ability of growers to sustainably meet future growth in fruit demand (12–14).

Numerous integrated pest management (IPM) programs have been developed to support pest-resistant varieties, biological control and mating disruption aimed at minimizing the use of insecticides (3, 5, 15, 16). Additionally, new technologies such as hail or shade netting are being considered as alternatives to insecticides. Netting can exclude insect pests from crops while minimizing risks to human health and the environment.

Hail netting has gained popularity in pome fruit orchards worldwide to exclude and protect against insect pests (17–19). Over the last 20 years, the suppression effect of exclusion netting used in pear and apple orchards for codling moth and brown marmorated stink bug (*Halyomorpha halys*) has been well documented in French and Italian orchards (20, 21). In Italy, researchers also found that hail netting suspended horizontally over apple orchard canopies was able to interfere with male codling moth’s ability to approach and mate with females, resulting in reduced fruit damage (22). Another Italian study in apple showed that single row hail netting effectively excluded a pest complex composed of leafroller moths (*Tortricidae latreille*), brown marmorated stink bug, and spotted-wing drosophila (*Drosophila suzukii* Matsumura), with no detrimental effect on fruit quality at harvest time (23).

In North America, several studies have documented the effectiveness of different types of exclusion netting for insect pests (17, 18, 24–26). In Quebec, Canada, a 5-year study documented the effectiveness of a single row, complete exclusion netting system that prevented damage from the apple maggot, codling moth, and the tarnished plant bug (*Lygus lineolaris*) (27, 28). A study in Washington found that codling moth could be excluded by using net cages placed around large orchard blocks (19, 29).

Previous studies have documented that both the apple maggot and codling moth are able to move through mesh sizes that are similar to those employed in hail netting (28, 30, 31). However, it is believed that the presence of netting interrupts mate-seeking behavior in the codling moth (22, 31). Similarly, netting may interrupt host-seeking behavior in apple maggot since they seek out trees and fruit primarily using visual and olfactory cues, but this has not been documented in the field (32–34). Most recently, Nelson (35) confirmed that large, highly motile insects such as codling moth, apple maggot, and others (including brown marmorated stink bug and Japanese beetle) are excluded by hail netting as well as small, 1x2 mm, insects such as minute pirate bugs (*Orius insidious*). It is believed that netting may pose a behavioral and/or visual obstruction to mate or food search behavior for a variety of insects (35).

In 2020, preliminary research in Minnesota explored the impact of using hail netting (DrapeNet®) in place of insecticides to exclude insect pests common to apples in the Upper Midwest. Preliminary data showed that hail netting significantly decreased codling moth by 92% and red-banded leaf roller by 78% (W.D. Hutchison, unpubl. data). The results of these preliminary observations spurred more rigorous on-farm field trials in 2021-2022. The trials were designed to explore the efficacy of using commercially available drape style white hail netting as a pest exclusion tactic on single rows of trees at two Minnesota apple orchards. In the study, the impact of hail netting was compared to conventional insecticide treatments by measuring pest populations, apple yield and apple quality. Researchers analyzed pest trap catches of adults, and concluded that codling moth and apple maggot were reduced by 94% and 96%, respectively, under the hail net treatment (without insecticide), compared to un-netted rows treated with insecticide sprays (36, 37). The efficacy of the netting for pest exclusion was greater than the control using a standard insecticide spray program.

**Figure 1:**
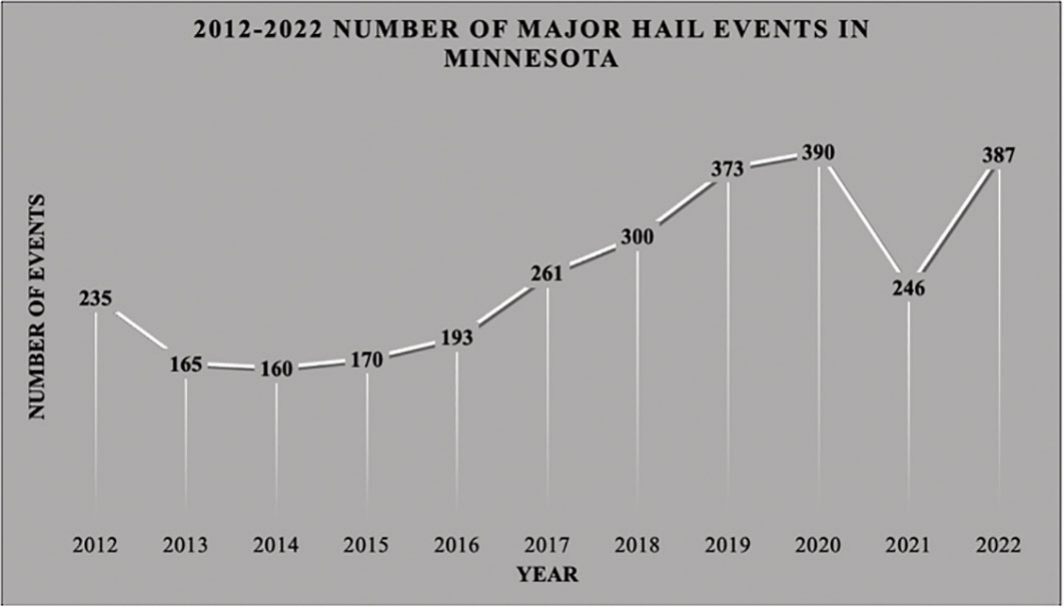
Number of major hail events in Minnesota (2012-2022), (hailstones >=2.5cm). Source: US Dept. of Commerce, National Oceanic and Atmospheric Administration (NOAA)’s National Weather Service, Storm Prediction Center.

In addition to controlling for pests, hail netting has the obvious advantage of protecting crops from hail and other deleterious environmental effects such as sunburn (e.g., 19, 26). These secondary benefits have important economic implications for growers in Minnesota and other Midwest states. During the past 10 years (2012-2022), Minnesota has ranked as one of the top ten U.S. states with major hail events (hailstones measuring ≥1” in diameter), averaging 262 events annually (**Figure 1**) (38). In 2022 Minnesota ranked 3^rd^ in the country with 387 major hail events (U.S. Dept. of Commerce, National Oceanic and Atmospheric Administration n.d.). Approximately 96% of all eligible agricultural crop acres were insured against hail in Minnesota that year with more than $361 million in losses paid out (39). Twenty-nine percent of surveyed Minnesota apple growers reported crop losses due to hail in 2022 (1). Hail can have a severe impact on apples by not only damaging the skin of maturing fruit, rendering it unmarketable for fresh sales, but also by causing structural and bud damage to trees thus reducing subsequent crop yield for up to three years (40). According to a 2017 study among New York, U.S. apple growers, fruit with hail injury resulted in a 96% decrease in crop value (41).

Federally subsidized hail insurance in the United States is a risk management strategy used by commercial apple growers to insure against hail losses, particularly in areas where the incidence of hail is historically high. Hail insurance in the United States is structured as an indemnity where apple quality, market price and production yield are factored into the insured crop value (42). Research suggests that insurance is the most financially efficient option for orchards with high hail risk, low apple yields and low value apple varieties (43). Alternatively, research has found that when first grade, high value apple varieties are considered, hail netting as opposed to insurance is a profitable risk management strategy for commercial growers (40, 43).

In addition to the economic benefits derived from the protection against insect pests and hail, netting may allow apple growers to capture price premiums for fruit produced without insecticides. Yue and Tong (44) studied Minnesota consumers’ willingness to pay for produce that was perceived “safe to eat,” “environmentally friendly” and “good for health.” In a series of hypothetical and non-hypothetical choice experiments, the researchers found that 66-89% of consumers, representing a diversity of socio-economic characteristics, were willing to pay a premium of up to 61% for environmental and food safety benefits conferred by organic production or practices. DrapeNet® is permissible under U. S. organic certification guidelines, while synthetic insecticides are prohibited by the National U.S. Organic Program (Pub. L. 101-624, title XXI, §2109).

In this study we explored whether hail netting, using the DrapeNet® treatment evaluated by Nelson et al. (37), is a cost-effective pest exclusion option for Midwest, U.S. apple growers compared to a traditional insecticide spray regimen as recommended for future research by Fornasiero et al. (21). The economic assessment was based on apple yield and quality observations reported by Nelson et al. (37). To our knowledge, there have been no published studies comparing economic costs and benefits of hail netting as an IPM strategy. The results of our study provide apple growers and other stakeholders with the information necessary to make objective, financially prudent IPM investment decisions for sustainable apple production.

## 2 Methods

In this section we describe the on-farm experimental design, data collection and economic assumptions as well as the methodologies used to assess the costs and benefits that accrue from using hail netting on apple orchards in the Midwest U.S. The primary economic assessment uses a partial budget (PB) methodology to assess the economic impact of changes in treatment costs, apple yield, apple quality, and changes in net income afforded by hail netting for pest control compared to the traditional use of insecticides. A secondary sensitivity analysis is used to assess the economic rigor of our PB results. Lastly, a deterministic analysis explores the added economic and risk management benefits that accrue to apple growers from controlling for hail damage.

### 2.1 Partial budget analysis

A PB methodology was used to empirically identify the most economically prudent pest control strategy for commercial apple orchards in the Midwest U.S. This methodology has been applied to explore difference between IPM and organic management systems for apples in New York (5) and to study economic benefits from a variety of mulching systems for watermelon in Alabama (45). More recently, the PB methodology was used to assess the costs and benefits of exclusion netting for pest control in Minnesota raspberries (46). The PB method is based on the principle that a minor change in operational management, such as the use of hail netting in place of insecticide sprays, can positively or negatively effect farm income. The operational change is considered preferable when the benefits of new practices or technology outweigh the costs (47–49).

The PB is structured as a table with two columns where the net change in income is represented as *NCI_0t_* = (*C_0_* + *R_t_*) - (*R_0_* + *C_t_*). The left-hand-side of the table has one column listing the negative economic effects associated with the added costs and reduced returns from the proposed change. In this case, the material and labor costs for installing, maintaining and removing the hail netting represent added costs (*C_t_*). Reduced returns are equal to the gross returns apple production using the conventional spray treatment (*R_0_*). The added costs and reduced returns are described in the PB as “added outflows.” Another column on the right-hand-side of PB table lists positive economic effects or “added inflows” which are represented by reduced costs associated with the original insecticide spray regimen (*C_0_*) and the added returns from marketable yields with the applied hail netting treatment (*R_t_*). The difference between the two columns, net change in income or *NCI_0t_*, indicates whether the proposed change (hail netting) will have a positive or negative effect on profitability when compared to the original treatment (insecticide sprays). When the change in income from the new hail netting treatment is greater than the conventional use of insecticide sprays, the *NCI_0t_* will be positive. Conversely, a negative *NCI_0t_* indicates that the original insecticide spray treatment is economically preferable to the proposed hail net pest management strategy.

The difference between added inflows and added outflows are used to estimate the benefit-cost ratio (BCR). This ratio is calculated as benefits divided by costs and is represented as BCR*_0t_* = (*C_0_* + *R_t_*)/(*R_0_* + *C_t_*). The BCR is a discrete way of identifying the most cost-effective control strategy and for prioritizing strategies (50). When the *BCR = 1.0*, the proposed change would break-even on expenses. A *BCR <1.0* indicates that the proposed change will lose money whereas *BCR >1.0* would generate a positive return on investments. The return on investment is represented as *P_0t =_ BCR_0t_ – 1.0* where *P_0t_* signifies the amount of profit generated for every $1.00 invested.

We explored two proposed changes or alternative strategies (*_t_*) in this study: hail netting (“Net-only”) and the combined use of hail netting plus insecticide sprays (“Net+spray”). The conventional insecticide sprays (“Spray-only”) represent the original baseline or “control” strategy (*_0_*) in our PB. **Table 1** shows the components of the PB by comparing the Net-only treatment against the baseline Spray-only treatment.

**Table 1:** Partial budget components comparing baseline Spray-only with Net-only.

Total Outflows	Total Inflows
Increased costs from Net-only	Increased revenue from Net-only
Tractor, 52 HP	SweeTango fancy, extra fancy apples
NetWizz	Minneiska apples
Tractor fuel	Utility apples
Hail netting	
Zip-ties	
Labor: panel stitching	
Labor: NetWizz set up and take down	
Labor: netting application and removal	
Labor: zip tie installation and removal	
Labor: harvesting and grading	
Reduced revenue from Spray-only	Reduced costs from Spray-only
SweeTango fancy, extra fancy apples	Tractor, 52 HP
Minneiska apples	Turbo Mist sprayer
Utility apples	Insecticides
	Water
	Hail crop insurance
	Labor: insecticide applications
	Labor: harvesting and grading
Total outflows = increased costs + reduced revenue	Total inflows = increased revenue + reduced costs

Net change in income = total inflows - total outflows.

Benefit-cost ratio = total inflows / total outflows.

### 2.2 Experimental design, data and economic assumptions

Data for the PB analysis come from field experiments at two commercial apple orchards owned and operated by the same family business - one in White Bear Lake, MN (Washington Co.; 45.108182, -92.950315) and the other in Preston, MN (Fillmore Co.; 43.682833, -92.074867). The field experiments were designed to compare the impact of hail netting for insect exclusion (Net-only) and the combined use of netting and insecticides (Net+spray) to a common insecticide spray regimen for apple orchards in the Midwest U.S. (Spray-only). The experimental design was applied at both farm study locations with a total of seven replications over two years: two replications in 2021 and 5 replications in 2022. Of these, three replications were installed in White Bear Lake (WBL), MN and four replications in Preston, MN. Treatments were randomly assigned to tree rows with four 30.48 m replications per row. For this study, it was assumed that each tree row measured 67.06 m with 30 rows/ha and 1,557 trees/ha. For netting exclusion effects on insect pest populations, see the Nelson et al. (37).

**Figure 2:**
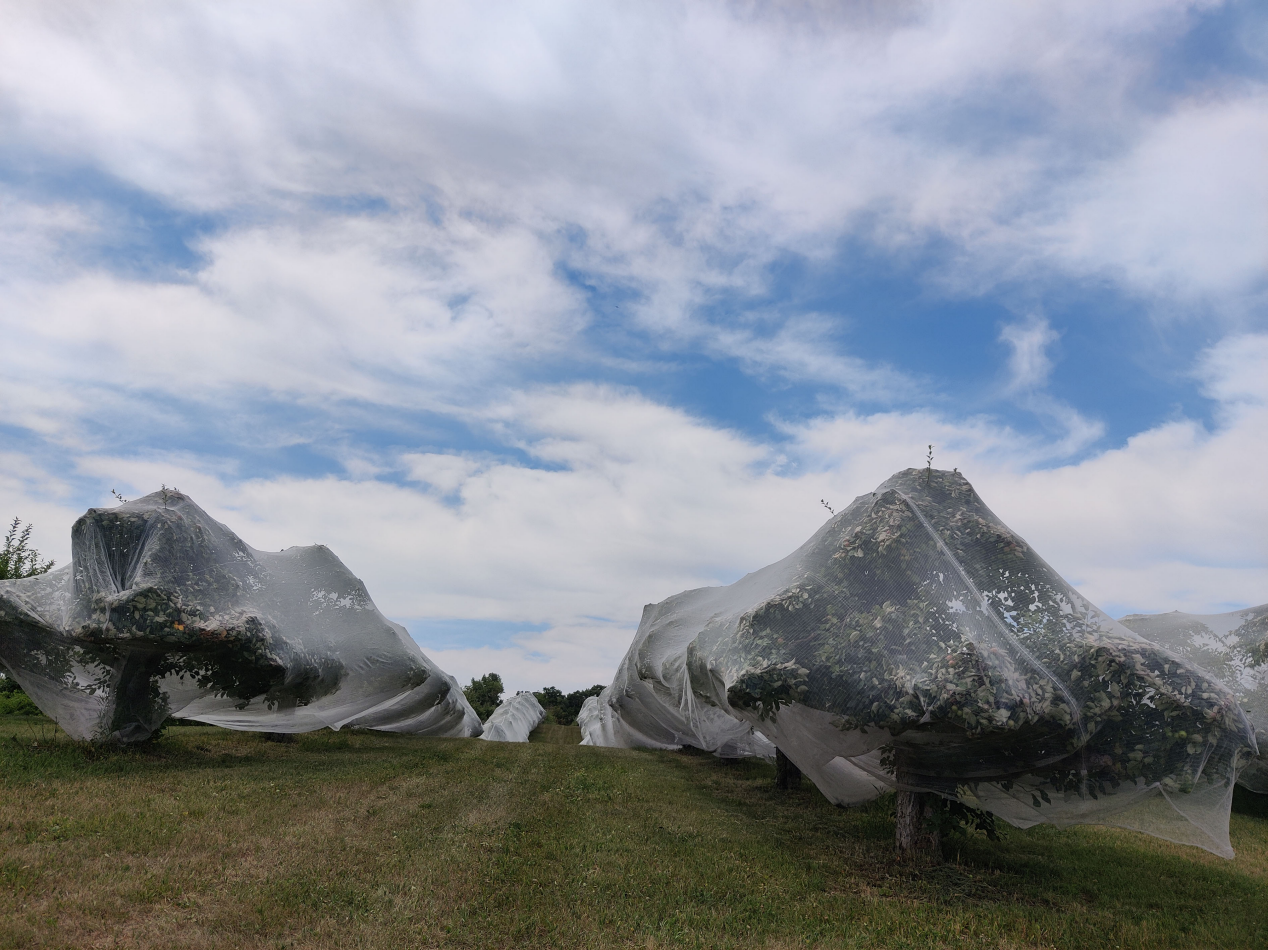
Hail netting applied to older, unsupported trees. Photo credit: Sally G. A. Nelson.

The orchards at both experiment locations were planted prior to the study with the SweeTango^®^ apple variety (2015 in White Bear Lake on G-11 rootstock, 2009 in Preston on B-9 rootstock). All trees in the study were of mature, fruit-bearing age. SweeTango^®^ is a managed (licensed) variety and was grown by 78% of Minnesota apple growers surveyed in 2023 (n = 51) (1). Trees were planted using high-density spacing (1.22 m between trees and 5.49 m between rows) and wire trellised supports (placed every 0.61 m). The economic analysis in this study assumes that the trellis structures were in place prior to netting installation and, therefore, were not considered part of the hail netting budget. Trellis structures are commonly used by growers who manage high-density orchards without netting. The grower in the experimental study, for example, maintained trellis supports throughout the majority of his orchards, including those areas where netting had not been previously applied. Moreover, the trellis system is not required for netting application; the grower in our study also successfully applied netting to older, larger trees without trellis supports (**Figure 2**).

Direct costs and budget assumptions that differentiate the hail netting and insecticide treatments to the study are summarized below and listed in **Table 2**. With the exception of diesel fuel and labor, all direct expenses calculated for 2021 were assumed to increase by five percent in 2022 due to inflation.

**Table 2:** Direct costs, 2021-2022 average^a^.

Item	Assumptions	2021-2022 average annual cost ($/hectare)
Tractor rental, 52 HP	Monthly rental is allocated across the entire orchard (28.33 hectares). Tractor usage varied by treatment and was charged accordingly.	50.05
Fuel for 52 HP tractor	Midwest US Energy Information Administration average cost of diesel fuel was $12.17 /liter in 2021 and $18.55/liter in 2022. The two-year average is reported on a per liter basis as fuel efficiency rates varied by task (spraying v. netting application and removal).	15.36
Netting Costs		
NetWizz Applicator	Cost of equipment is $18,000 with a salvage value of $1,800 after 20 years. The annual straight-line depreciated value was allocated across the entire orchard (28.33 hectares).	28.58
DrapeNet® Netting	Netting sold in bundles measuring 99.97 meters long x 7.01 meters wide for a cost of $420/bundle. The bundle cost was allocated over 12 years using straight-line depreciation and an assumed salvage value of $0.00.	760.81
Zip Ties	Ties cost $0.01 each and were placed every 1.22 meters.	27.53
Insecticides		
Turbo Mist Sprayer, 300 gal.	Cost of equipment was $15,000 with a salvage value of $1,500 after 20 years. The annual straight-line depreciated value was allocated across the entire orchard (28.33 hectares).	24.41
Belay (clothianidin) – insecticide	The 2-year average cost was $82.15 per liter, applied 1X in May each year at rate of ~0.44 liters per hectare.	36.00
Agrimek (abamectin) - insecticide	The 2-year average cost was $142.80 per liter, applied 1X in May at rate of ~0.29 liters per hectare.	41.72
Rimon (novaluron) - insecticide	The 2-year average cost was $76.25 per liter, applied 1X in June at a rate of ~ 0.24 liters per hectare.	18.30
Assail (acetamiprid) - insecticide	The 2-year average cost was $176.42 per liter, applied 1X in July and 1X in Aug at a rate of ~0.44 liters per hectare each time.	155.24
Trellis Costs		
Post pounder	The cost of equipment was $10,000 with a salvage value of $1,000 after 20 years. The annual depreciated value was allocated across the entire orchard (28.33 hectares).	6.43
Wood posts	Treated 14’X6”wood posts cost $15 each and were spaced 5.49 meters apart along each row. An additional post was placed at the end of each row. The posts were assumed to have a 15-year useful life with zero salvage value.	414.96
High tensile wire	High tensile 12.5-gauge wire cost $0.21 per meter and was assumed to have a 15-year useful life with zero salvage value. Wire strung every 0.61 meters horizontally and attached to wood posts with staples.	139.14
Strainers	Strainers cost $3.00 each and were assumed to have a 15-year useful life with zero salvage value. One strainer per wire placed at the end of each row.	29.64
Trellis clips	Clips cost $0.67 each and were assumed to have a 15-year useful life with zero salvage value. Five clips were used to secure each tree to the wires.	59.77
Staples	Grower estimated the cost of staples at cost at $44.93 per row. We assumed that the staples have a 15-year useful life with zero salvage value.	88.78

^a^Cost estimates provided by co-author J. Jacobson, based on recent costs incurred at Pine Tree Apple Orchard, White Bear Lake, MN, U.S., as well discussions with other MN growers, 2021-2022.

**Figure 3:**
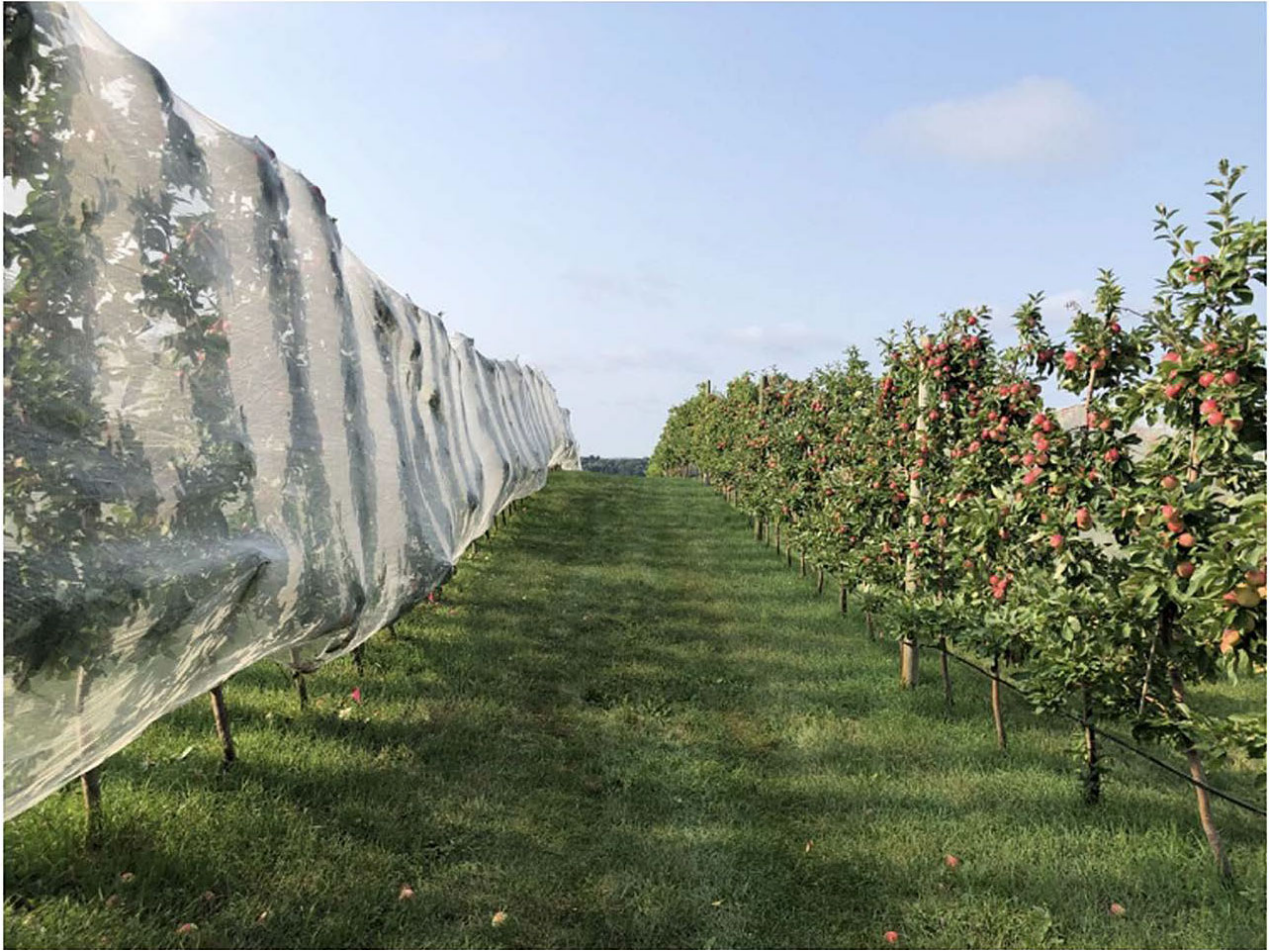
Hail netting applied to single row trellised trees on left-side of photo. Photo Credit: Gigi DiGiacomo.

For the Net-only treatment, direct costs included netting, zip ties for netting attachment, two-month tractor rental, tractor fuel, and labor for netting application and removal as well as the annual depreciated cost of the netting applicator. Netting (**Figure 3**) was applied to the tree rows for the Net-only treatment in spring after petal fall, ensuring that pollination of apple blossoms was complete (WBL: 26 May 2021, 6 June 2022; Preston: 1 June 2021, 8 June 2022). The netting was removed after harvest in the fall (WBL: 1 September 2021, 2 September 2022; Preston: 31 August 2021; 31 August 2022). The 6 X 1.8mm white netting (DrapeNet^®^ USA, Prosser, WA) is approved for organic certification and was valued annually at $761/ha using a 10-year depreciation rate with no salvage value. The cost of netting included a one-time labor expense of $5.68/ha to sew panels to the appropriate length needed for each planted row.

**Figure 4:**
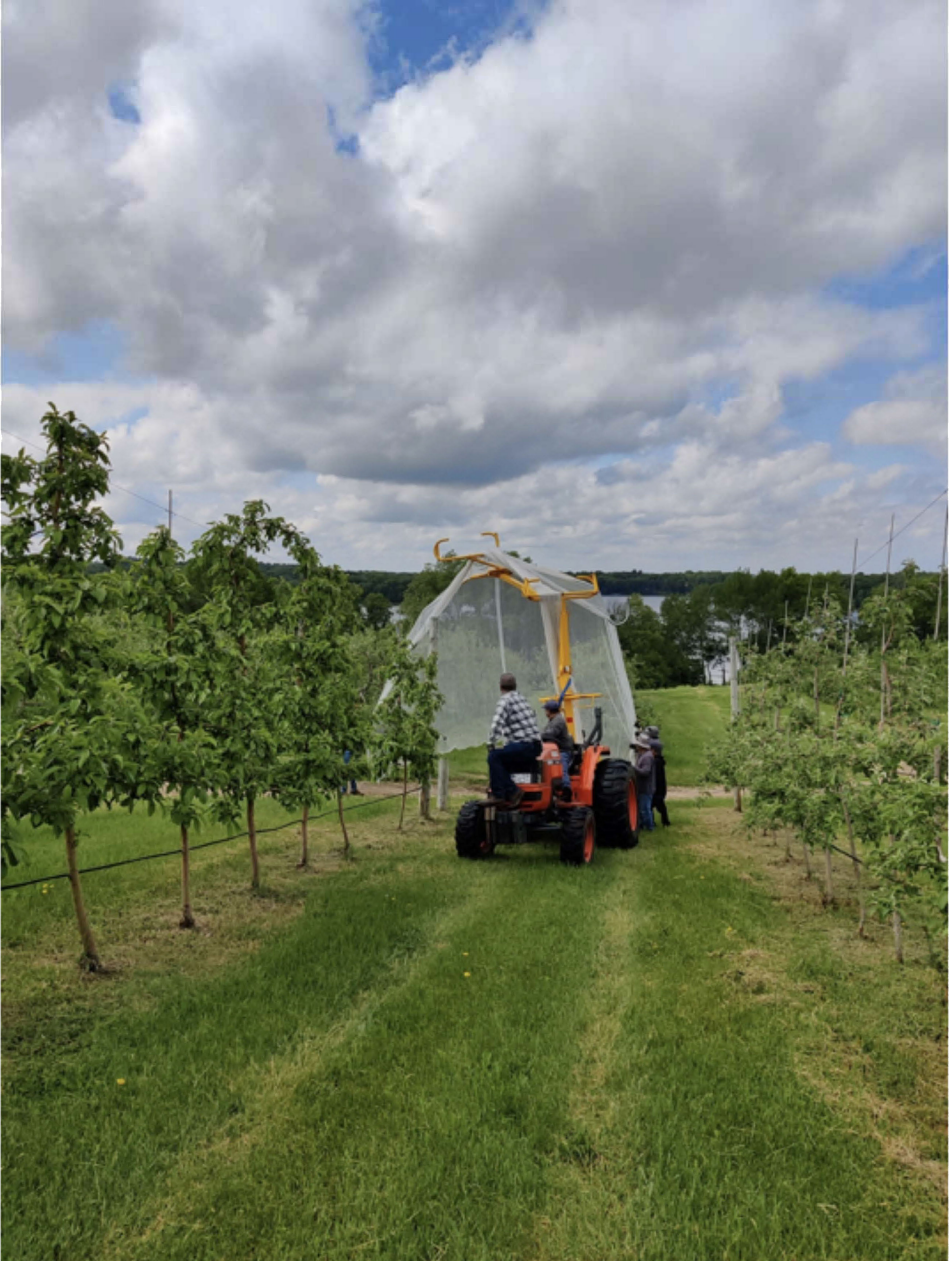
Hail netting applied with Net Wizz applicator. Photo credit: Sally G. A. Nelson.

The net applicator, NetWizz (DrapeNet® USA, Prosser, WA), was valued new at $18,000 and annualized for this study at $28.58/ha using straight line depreciation over 20 years with a 10% salvage value. The NetWizz was attached to the back of a 52-HP tractor which allowed the netting to unroll and drape over a single row of trees using the traditional “over tree” method (**Figure 4**). Branches were pruned on either side of the trees as part of a regular winter pruning schedule to approximately 0.50 m long to make this system conducive to machine application of hail netting. All high-density trellis trees, including those receiving the Spray-only treatment, were pruned and thinned to an equal extent. Netting was secured to the base of trees using zip ties (Fleet Farm) every 1.22 m. Zip ties were valued at $0.01 each or $27.53/ha. The netting remained on the trees until harvest, covering the trees throughout fruit development. Netting was removed prior to anticipated harvest using the NetWizz. Hail netting was installed and removed by the grower using standard procedures for commercial orchards, without any modifications for the purposes of the study.

According to grower records, netting installation and netting removal required five people and a combined total of 211 minutes (3.52 hr/ha using a 52-HP tractor assuming standard travel rates). The netting for this project had been used with success by the grower since 2018, thus the application and removal rates are considered efficient for the industry. Additionally, orchard staff spent 55.45 min/ha to assemble and dissemble the NetWizz applicator each time it was used. Labor was valued at the 2-year average rate of $16.52/hr for spring netting application and $17.49/hr for fall netting removal. Labor rates were calculated using the National Agricultural Statistics Service (NASS) farm labor rates for Lakes states in April 2021-2022 and October 2021-2022 (USDA 51, 52).

The 2021-2022 tractor rental rate was provided by the grower and averaged $50.05/ha/month when allocated over 28.33 ha. The tractor was required for two months each year (May and September) for the Net-only treatment during application and removal. Tractor use for harvest and other orchard activities were equivalent across treatments and thus were not included in direct costs for the Net-only or any other treatments in the PB analysis.

For the Spray-only treatment, direct costs included the annualized cost of an insecticide sprayer, insecticides, tractor rental, tractor fuel, labor, and hail insurance. The grower in our study made a total of five insecticide applications for un-netted trees each year, in 2021 and in 2022, at each study location. The first sprays (Belay at 0.44 l/ha and Agrimek at 0.29 l/ha) coincided with petal fall. Additional insecticides were applied in late spring, Rimon at the first flight of codling moth (May 18, 2021 and June 1, 2022 at 0.24 l/ha), and in summer, Assail for apple maggot (July 5, 2021, August 1, 2021, July 13, 2022 and August 4, 2022 at 0.44 l/ha each year). Together, the five insecticide applications were valued at $251/ha using 2021-2022 grower rates and market values. Apple growers in Minnesota typically apply seven insecticidal active ingredients and spray seven or more times, on average, each season (1). However, the grower in our study has been rigorous about pest control, including the application of netting since 2018, and has observed relatively low background pest densities, particularly for codling moth and apple maggot (measured using traps). Sprays were only administered when pest action thresholds were reached.

Direct equipment costs for the Spray-only treatment were estimated using monthly tractor rental rates and the depreciated cost of a new 300-gallon Turbo Mist air blast sprayer. The sprayer was estimated at $15,000 employing market rates and annualized at $24.42/ha using straight line depreciation over 20 years with a 10% salvage value. Based on insecticide spray schedules, the tractor was required for four months (May-August) for the spray treatments and was valued at $50.05/ha/month - the same rate used for the Net-only treatment.

Diesel fuel expenses were compiled based on tractor travel rates provided by the grower: 80.47 m/min traveling at a speed of 4.82 km/hr resulting in an average rate of 0.11 l/min. Annual diesel fuel rates for the Midwest U.S. averaged $12.17/l in 2021 and $18.55/l in 2022 (53). Applying these rates, we estimated annual average tractor fuel costs at $15.36/ha over the 2-year period.

Harvest and grading labor for all treatments were tracked by research staff for the study period and corroborated with the grower to verify that rates were efficient or near equivalent to rates observed for orchard staff. Harvest rates were estimated at 327 kg/hr and valued using the NASS farm labor rates for Lakes states. The hourly rates paid to field workers compiled by NASS for October 10-16, 2021 and October 9-15, 2022 used in this study were $16.75/hr and $18.26/hr, respectively (51, 52).

Hail insurance for the Spray-only treatment was estimated using the USDA Risk Management Agency “Cost Estimator” for APH90 with an assumed 75% loss rate for fresh, high value apples in Washington and Filmore counties (42, 54). The estimated annual average cost for federally-subsidized hail-related crop insurance was $1,166/ha.

For the Net+spray treatment, all direct costs for the individual Spray-only and Net-only treatments were combined with the exception of tractor and fuel needs. These were adjusted to account for overlapping use; tractor rental and fuel use was assumed for six months (May-October).

Club marketability ratings were applied to grade the apples and measure apple quality and yield. Ratings used were: extra fancy and fancy SweeTango^®^, Minneiska, utility, and cull. Club members are licensed to grow managed varieties like SweeTango^®^ and determine quality standards for marketability (55). Only apples that graded out at extra fancy and fancy are permitted to use the branded name SweeTango^®^ and were valued at $5.74/kg in 2021 and 2022 by the grower. This price reflects a premium of approximately 25% above retail prices for conventional high-value apple grades and a discount of approximately 25% from organic retail apples for a similar high-value variety (56). Therefore, it can be assumed that the grower has captured a portion of the market premium available through direct marketing. Varietal seconds sold fresh are called “Minneiska” and were priced at $3.45/kg each year. Other seconds that were not fit for the fresh market were labeled “utility” and used or sold for processing (e.g., cider and apple pie). Utility grade apples were valued at $0.55/kg in 2021 and 2022. Apples that did not qualify as marketable (either for fresh or processing) due to any break in the fruit skin were culled and assigned no market value. Varietal seconds and utility-grade apples were also valued using prices provided by the grower; prices were verified with secondary data using published rates where available (**Table 3**).

**Table 3:** Apple Prices, based on 2021-2022 averages for Minnesota and Midwest, U.S. markets^a^.

Variety & grade	Grower-reported price ($/kg)^b^	USDA-AMS organic retail price ($/kg)^c^
SweeTango™	5.74	7.05
Minneiska	3.45	4.23
Utility	0.55	0.68

a/ Grower prices for all grades were supplied by J. Jacobson, Pine Tree Apple Orchard, White Bear Lake, MN, U.S. The price ratios (grade price/ SweeTango™ price) for all apple grades provided by the grower were applied to premium grade prices from other sources to arrive at second grade (Minneiska) and utility grade price estimates. Price ratios used: SweeTango = 100%; Minneiska = 60%; utility = 10%.

b/ Represents prices reported by J. Jacobson, Pine Tree Apple Orchard, for pre-picked apples marketed retail through the farm store and to schools as well as wholesale for on-farm processing in White Bear Lake, MN, U.S.

c/ Represents weighted average organic prices for similar high-value grade apple (Honeycrisp) reported by 146 retail grocery stores in the Upper Midwest by the USDA Agricultural Marketing Service.

Costs associated with ongoing tree maintenance, fertilization, disease management and other orchard management costs for all treatments were assumed to be the same across treatments and therefore are not included in the PBs. Fixed costs such as buildings and land as well as other items that remained unchanged also were not included in the PB as is standard for this methodology (57, 58). Accordingly, our analysis did not include land, material or operating costs associated with trees, tree establishment, trellis supports, fertility management and other factors that remained unchanged between the treatment comparisons.

### 2.3 Marketable yield analysis

Yield data from the on-farm trials were obtained and applied in a statistical analysis to study any significant differences in marketable yield and quality for the PB (37). During the two-year study period, 1,350 apples were sampled and graded following SweeTango^®^ club specifications on the basis of: percent red skin color, size, deformity, and blemishes. For the PB analysis, we assumed that marketable yields observed in the sample were consistent throughout the orchard.

Statistical analyses for marketable yield were conducted using R Studio v4.2.2 (59; package, nlme) (60) for a linear mixed effects model: with a fixed effect of treatment (Spray-only, Net-only, Net+spray) and random effects of replication nested within year. Two separate analyses were used to model the yield for the fancy quality fruit grade (SweeTango^®^) and all marketable fruit (SweeTango^®^ + Minneiska + utility grade) fruit within the different pest management treatments. The marketable yield for each treatment was calculated by multiplying the total yield per sample by the percentage of fruit in that sample that were in each fruit quality category (fancy, Minneiska, utility, etc.). Graphical inspection of residual plots allowed determination that analytical assumptions of both models were met.

### 2.4 Sensitivity analysis

Sensitivity analyses can be used to measure business exposure to risk, to identify opportunities and to test the robustness of results for feasibility and decision-making (61). A sensitivity analysis was performed in this study by varying marketable yield, apple prices received by the grower, and the number of insecticide applications for both netting treatments.

The change in marketable yield was determined by substituting the numerical yields observed during the field study for the two netting treatments in place of the statistically significant yield results. Yield differences were expected to impact added costs and added benefits. On the cost side, differences in harvest and grading labor were accounted for as this expense is proportionate to yield. On the benefit side of the PB table, increases in the observed marketable yield were anticipated to increase added benefits.

Next, we explored the impact on net changes in income by varying apple prices to include organic premiums for the Net-only treatment. Organic prices were not applied to the Spray-only or the Net+spray treatments as research suggests consumers are only willing to pay a substantive price premium for produce grown without insecticides (44). Weighted average organic apple prices were compiled from Midwest retail grocery stores by the USDA-AMS in 2021-2022 (**Table 3**). USDA-AMS does not report prices for SweeTango^®^, so prices for an equivalent high-value apple variety, Honeycrisp, were applied in the sensitivity analysis. We calculated the percent difference in apple values for each grade as provided by the grower and applied these same differences to the USDA-AMS premium grade apple prices to infer an estimated equivalent for the premium marketable grade (SweeTango^®^), secondary marketable grade (Minneiska) and the processing grade (utility). The change in prices for the Net-only treatment were expected to improve added inflows or benefits in the PB analysis.

Finally, the number of insecticide applications were assumed to increase from five to seven in the Spray-only scenario by adding one supplementary application of Rimon for codling moth and one additional application of Assail for apple maggot. As mentioned previously, apple growers in Minnesota typically treat apples seven or more times, on average, with insecticides each season (1). In other locations, it is not uncommon to spray 10 times per season for apple maggot alone (2–5). Added insecticide costs for the Spray-only treatment included the cost of the insecticides and tractor fuel for the two additional spray applications as well as labor for spray preparation, insecticide application and equipment cleanout.

### 2.5 Deterministic risk analysis

A deterministic risk analysis, where all assumed risks are known, was computed for different marketable apple grade and hail loss scenarios. This analysis assesses the minimum hail loss required to justify netting in absence of insurance indemnities by comparing the equivalent increased returns per hectare (gross revenue) under different yield loss scenarios. Given that we were unable to identify secondary data for rates of insurance use and hail-related losses specifically for apple growers in the Midwest, we assumed a range of hail-related losses ranging from 0%-100% of the annual fresh-market SweeTango^®^ and Minneiska apple yield for the analysis.

## 3 Results

The results of our PB analysis suggests that hail netting is a financially competitive pest management alternative to insecticide sprays for Minnesota apple growers. Over the two-year study period comparing hail netting and insecticide treatments, the PB indicates marginal cost savings from the Net-only treatment without any reduction in apple yield or quality. These results suggest that hail netting is an economically viable pest control option for growers looking to reduce or eliminate insecticides in their orchards.

### 3.1 Apple yield and quality

Total and marketable apple yields were numerically higher for both the Net-only and the Net+spray treatments compared to the Spray-only treatment (**Table 4**). Total apple yield (22,164 kg/ha) and marketable apple yield (18,732 kg/ha) were 27% and 32% higher, respectively, for the Net-only treatment compared to the Spray-only control treatment. Similarly, the Net+spray treatment produced total apple yields (22,855 kg/ha) and marketable apple yields (21,107 kg/ha) that were 31% and 49% higher numerically, respectively, compared to yields from the Spray-only treatment.

**Table 4:** Apple yield under different pest management treatments, 2021-2022 averages.

	Spray-only (Control)	Net-only (Treatment 1)	Net+spray (Treatment 2)
2021-2022 averages, kg/ha (± std dev)			
SweeTango^®^	6,400 (± 6,085)	8,734 (± 6,241)	10,423 (± 4,862)
Minneiska	5,417 (± 2,595)	8,077 (± 2,874)	8,694 (± 4,215)
Utility	2,350 (± 2,425)	1,921 (± 773)	1,989 (± 1,998)
Marketable Yield	14,167 (± 7,839)	18,732 (± 7,313)	21,107 (± 6,686)
Unmarketable Culls	3,285 (± 1,203)	3,432 (± 1,884)	1,748 (± 755)
Total Yield	17,452 (± 5,539)	22,164 (± 9,563)	22,855 (± 7,194)

In addition to overall improvements in total and marketable yield, apple quality appeared to improve under the netting treatments with the fancy SweeTango^®^ apple grade accounting for a larger share of numerical marketable yield for the Net-only treatment (36%) and the Net+spray treatment (63%) compared to the Spray-only treatment. Similarly, the Net-only and the Net+spray treatments averaged a 49% and 60% improvement in the numerical Minneiska apple grade, respectively, compared to the Spray-only treatment over the two-year period studied (**Table 4**).

Despite differences in numerical yield, the results of the linear mixed effects model suggest there were no statistically significant differences in total and marketable yield between treatments (**Table 5**). Only the Net+spray treatment generated statistically significant quality differences for the fancy SweeTango^®^ apple grade (p = 0.0083) (**Table 5**). However, when looking at all marketable yields combined (SweeTango^®^ + Minneiska + utility grades), the statistical model indicated no significant differences; there were no strong trends in the yield data to indicate any differences amongst treatments. For this reason, we assumed no difference in yield for the PB analysis; Spray-only marketable yields were applied to the Net-only and Net+spray treatments thus returning no differences in gross income from the Spray-only treatment and the netting treatments.

**Table 5:** Generalized linear mixed effects model results.

	Std. Error	D.f.	p-value
SweeTango®			
Spray-only	2.73352	10	0.0013
Net-only	1.07204	10	0.8708
Net+spray	1.07204	10	0.0083**
SweeTango® + Minneiska			
Spray-only	3.79501	10	0.0002
Net-only	2.81665	10	0.5283
Net+spray	2.81665	10	0.1553
All marketable†			
Spray-only	3.92324	10	0.0001
Net-only	2.98894	10	0.3077
Net+spray	2.98894	10	0.3183

† All marketable apples include: SweeTango™ + Minneiska + utility grades.

*p-value significant at .05; **p-value significant at .00.

### 3.2 Treatment costs

Turning to estimated expenses, we found that the Net-only treatment was the least expensive of all options considered; increased costs for the Net-only treatment totaled $2,329/ha with netting, zip ties, equipment purchases, equipment rental and labor included (**Table 6**). Comparatively, reduced costs representing expenses for the Spray-only treatment, totaled $3,271/ha. Lower costs for the Net-only treatment were explained in large part by the savings from foregone federally-subsidized hail insurance. These savings, and hence the spread between the Net-only and Spray-only costs, would have been larger had the full cost of hail insurance been borne by the grower.

**Table 6:** Partial budget comparing Net-only and Net+spray treatments to Spray-only control treatment assuming no difference in yield, 2021-2022 averages.

	Net-only ($/Hectare)	Net+spray ($/Hectare)
Results w/Observed Field Trial Yields		
Increased costs	2,329	3,448
Reduced returns	56,715	56,715
Total outflows	59,044	60,164
Increased returns	56,715	56,715
Reduced costs	3,271	3,271
Total inflows	59,986	59,986
Net change in income	942	-178
Benefit-cost ratio	1.02	1.00

Increased costs for the Net+spray treatment were $3,449/ha (**Table 6**); equal to the Spray-only treatment and Net-only treatment costs combined with some realized savings in hail insurance and tractor rental. There were marginal cost differences between the Spray-only and Net+spray treatments. The Spray-only treatment realized $3,271/ha in costs.

### 3.3 Change in net income

Any differences in the change in net income for the PB analysis were due solely to differences in costs given that marketable yield, apple prices, and hence, gross income, under all treatments were assumed to be the same. Observed differences in the reduced costs generated a modest positive change in net income for the Net-only treatment of $942/ha with a BCR of 1.02 compared to the conventional Spray-only control (**Table 6**). In other words, for every $1 invested in the Net-only treatment, the return on investment was just above break-even.

The Net+spray treatment incurred a slight loss compared to the Spray-only treatment when the same Spray-only yields and observed grower prices were applied. The change in net income from the Net+spray treatment compared to the Spray-only treatment was -$178/ha with a BCR of 1.00 (**Table 6**). Significant marketable yield benefits would have been needed to offset the higher costs of the combined netting and spray treatments.

### 3.4 Sensitivity analysis

We explored how changes in marketable yield, apple price, and the number of insecticide applications affected the robustness of our PB results by applying a sensitivity analyses to the three different variables independently. First, the Spray-only yields used in the original PB analyses for all treatment options were replaced with the numerical yields observed in the field trials for the netting strategies. Numerical marketable yields for the Net-only and Net+spray treatments were 32% and 49% higher, respectively, than the numerical Spray-only yields observed in the field trials (**Table 4**). Applying observed numerical yields, the net change in income for the Net-only treatment compared to the Spray-only was $22,978/ha with a BCR of 1.39 (**Table 7**). The BCR suggests that for every $1.00 invested in the Net-only strategy, $0.39 of profit would be generated. Similarly, the Net+spray treatment, which produced the highest numerical yields thanks to the larger share of high-quality SweeTango^®^, generated a net change in income equal to $33,735/ha in the PB with a BCR of 1.56 (**Table 7**).

**Table 7:** Sensitivity analysis: Net change in income under different yield scenarios and market prices.

	Net-only, $/ha (BCR)	Net+spray, $/ha (BCR)
Baseline^a,b^	942 (1.02)	-178 (1.00)
Increased (7) insecticide sprays^a,b^	1,261 (1.02)	-178 (1.00)
Increased (observed) yields^a,c^	22,978 (1.39)	33,735 (1.56)
Increased (organic) prices^b^	24,595 (1.51)	Not applicable

a/ Grower prices applied to all treatments and apple grades. Grower prices were supplied by the grower for SweeTango™, Minneiska and utility grades and are listed in **Table 2**.

b/ Spray-only yields assumed for all treatments; no differences in yields. Treatment yields are listed in **Table 4**.

c/ Yields observed in field trials for each treatment are assumed. Treatment yields are listed in **Table 4**.

d/ Organic prices were applied to the Net-only treatment as it is assumed that Net-only apples could be marketable as “organic” as they were produced without the use of insecticides. Organic prices were drawn from the USDA-AMS “Weekly advertised fruit & vegetables retail prices” reports and are listed in **Table 2**.

Next, we varied the prices received by the grower in the Net-only treatment while continuing to assume no difference in yield between the Spray-only and Net-only treatments and the original pesticide spray regimen observed. Organic price premiums were applied to the Net-only treatment to reflect consumer preferences for fruit grown without insecticides. Weighted average organic retail prices for high-value apples, $7.05/kg, compiled by the USDA-AMS, were 23% higher than the grower-reported prices of $5.74/kg (**Table 3**). Under the organic price scenario, the net change in income for the Net-only treatment increased from $942/ha to $24,595/ha (**Table 7**). Similarly, the BCR improved from 1.01 to 1.51, all other things being equal, making the organic price scenario for the Net-only treatment considerably more profitable than the Spray-only or the Net+spray treatments valued at grower-reported prices.

Finally, the number of insecticide applications were increased conservatively from five to seven in the Spray-only scenario by adding one additional spray of Rimon for codling moth and one additional spray of Assail for apple maggot. No changes in yield or price were assumed. The two additional insecticide treatments increased the Spray-Only costs by $319/ha from $3,271/ha to $3,590/ha. With this 10% cost increase, the Net-only treatment realized a change in net income equal to $1,261/ha over the Spray-only treatment (**Table 7**); there was no significant change in the BCR. No differences were observed for the Net+spray treatment as we assumed that the same costs would accrue to this treatment option as with the Spray-only option (**Table 7**). Thus, with the addition of two more insecticide applications, the Net-only treatment would become slightly more attractive, financially, for Midwest apple growers compared to the Spray-only and Net+spray pest management strategies, particularly for those who operate on a larger scale and can take advantage of marginal gains.

### 3.5 Deterministic analysis

**Figure 5:**
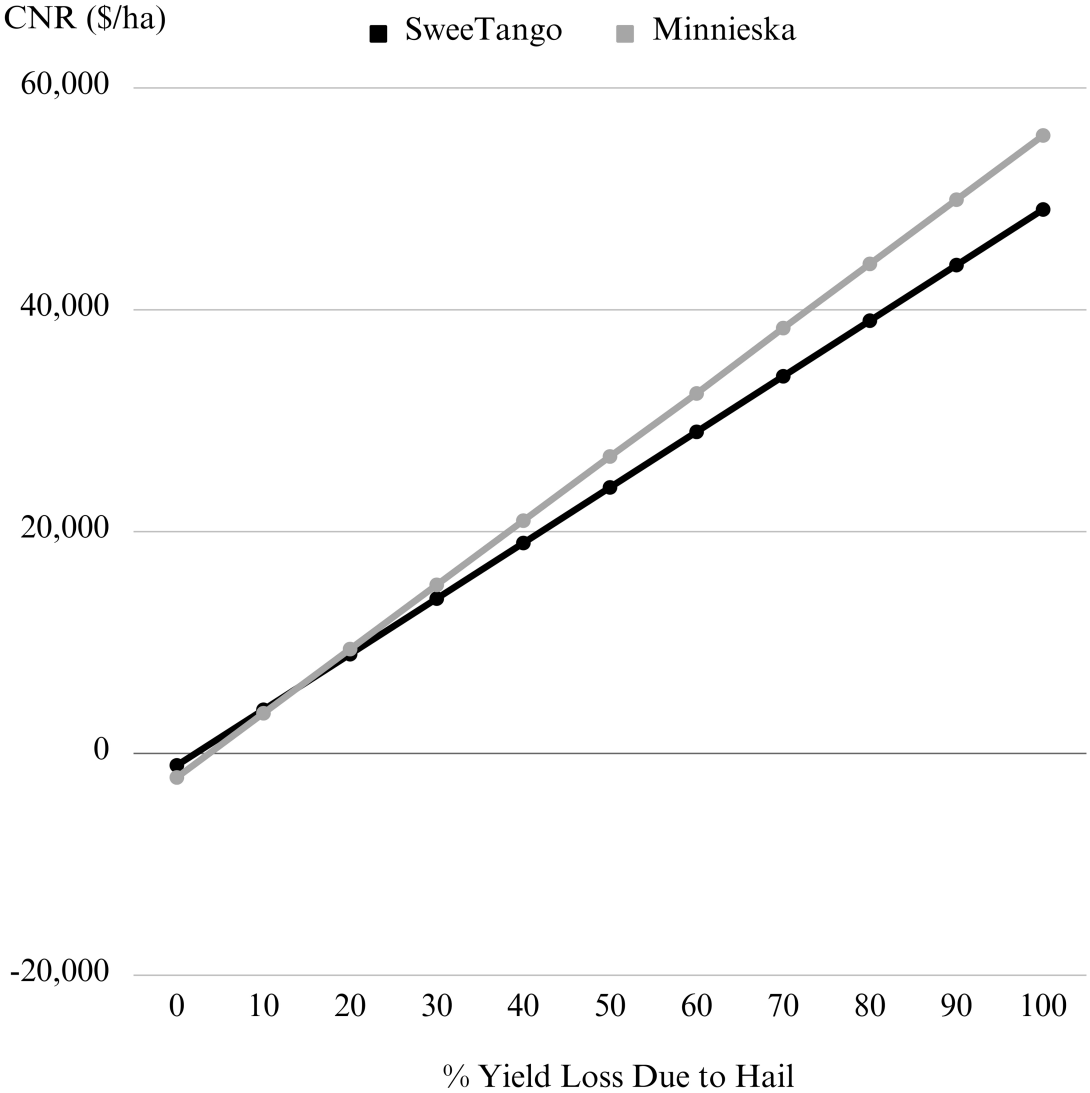
Change in net return (CNR) for hail netting under different yield loss scenarios.

Lastly, results from the deterministic analysis indicated that the hail netting more than paid for itself when hail-related yield losses were 5% to 10% of marketable apples (**Figure 5**). We assumed Spray-only yields and grower prices for the deterministic analysis and found that hail storms producing a 5% yield loss returned $443/ha above the netting costs for SweeTango^®^ apples and -$459/ha for the Minneiska grade apples. During a 10% loss event, the averted hail damage returned $2,280/ha above the netting costs for SweeTango^®^ apples and $476/ha for the Minneiska apples. Finally, in the event of extreme hail storms, when 100% yield loss was assumed for un-protected trees, the netting investment would have returned $35,340.21/ha for the SweeTango^®^ apples and $17,295.86/ha for the Minneiska. It is only in the complete absence of hail events - when yield losses were zero - that the full costs of netting were incurred as lost income, -$1,393/ha regardless of apple variety.

## 4 Discussion

Netting studies published thus far for apple fruit have focused on insect pest exclusion benefits in Europe (20, 22, 23) and North America (17, 19, 24, 31). However, as noted by Onstad and Crain (62) and Fornasiero et al. (21), the vast majority of evaluations do not include formal economic analyses. One exception is the economic assessment of IPM and organic pest management for apples in New York where a partial budget approach was also used (5). However, to our knowledge previous studies designed to assess the economic value of hail netting for IPM purposes have not yet been published.

We compared exclusion netting treatments, that used DrapeNet® designed for hail, to a traditional insecticide spray regimen at two Minnesota orchard locations in 2021-2022. The hail netting allowed for a significant reduction in insecticide applications with no impact on fruit quality or yield and marginal income benefits.

In the PB, we assumed no difference in marketable yields between treatments based on the statistical results and supported by Aćimović and Leffelman (26). Under this assumption, no direct income benefits accrued as yields, grower prices and, consequently, gross income were equal across all treatments. We did, however, observe minor benefits to the Net-only treatment from cost savings. Direct costs totaled $2,329/ha for the Net-only treatment compared to $3,271/ha for the Spray-only treatment - producing a 29% savings equal to $942.01/ha for the netting option. The Net-only treatment broke-even on investment costs when yield and apple prices were treated equally across treatments.

When numerically-higher yield observations from the study were applied to the netting treatments, the change in net income rose from $942/ha to $24,595/ha for the Net-only treatment (**Table 7**). The application of numerically higher yields is justified given findings by Candian et al. (18) who observed significant positive post-harvest differences in pome fruit from netted plots compared to fruit treated with insecticides. Fruit from un-netted plots treated only with insecticides were significantly more likely to develop post-harvest rot compared to trees treated only with netting (18). While Nelson (35) did not study post-harvest fruit rot at harvest or after storage to determine the impact of netting on apple yield or quality, we assumed that the results from Candian et al. (18) would apply. Therefore, the decision to substitute the numerically-higher Net-only yields for the sensitivity analysis has merit and further supports the study’s conclusion that netting confers economic benefits when compared to insecticide treatments for apples in the Midwest, U.S.

The decision to apply organic price premiums to apples harvested from the Net-only treatment in the sensitivity analysis had a substantial impact on the financial outcomes from netting. The change in net income for the Net-only treatment when organic prices were assumed rose from $942.01/ha to $24,595.35/ha (**Table 7**). Short of knowing the specific price premium Minnesota consumers would be willing to pay for apples grown without insecticides, we applied the full organic premium to the Net-only treatment due to the absence of insecticide use (**Table 3**). The USDA-AMS organic premium applied in this study was 44% above the weighted average conventional Midwest retail apple price and 17% higher than the reported weighted average Minnesota grower price. It is reasonable to assume a 17% to 44% organic price premium given previous work by Yue and Tong (44) who found that consumers in Minnesota generally were willing to pay an additional 61% (above conventional prices) for environmental and food quality benefits. In reality, however, if conventional (non-organic) fungicides and fertilizers were used in the orchard along with netting, the apples could not be sold as “certified organic.” Thus, other management changes would be required to qualify for certified organic price premiums. Since the full production costs were not considered as part of this study, we did not estimate how large an impact other organic management changes might ultimately have on net costs and benefits. This should be explored further in future research, but it is safe to assume that additional costs would not outweigh the added income benefits observed ($24,595/ha). Taylor and Granatstein (63) compare organic and conventional apple production costs for Washington, U.S. growers and found a net difference of 13% or $2,307/ha (adjusted for inflation) in variable costs.

The final consideration explored in the sensitivity analysis concerned the number of insecticide applications used in the Spray-only treatment. We explored a moderate increase in the number of insecticide spray treatments from 5 to 7 annually and found that the difference in costs had a minimal impact on the overall change in net income, particularly for small-scale growers with limited acreage. The real benefits of netting under the increased spray scenario would have accrued to larger-scale growers who could take advantage of marginal income benefits, particularly if we had assumed 10 insecticide applications as is common for apple management in other areas of North America.

The current analysis makes a significant contribution to IPM literature by quantifying the economic costs and benefits associated with the adoption of hail netting for pest control. Results indicate that under conservative conditions (where no differences in marketable yield or market price were assumed, insecticide applications were relatively low, and federally-subsidized rates of hail insurance were applied) hail netting is a cost competitive IPM alternative to conventional spray programs for Midwest apple.

Growers managing an orchard that is completely (or nearly) netted, may consider reducing insecticide applications for major pests without sacrificing income and, at the same time, garnering the secondary financial benefits associated with crop protection from hail. These observations have important global implications for fruit production. Stratton et al. (64) recommend the development of innovative, multi-dimensional production strategies that include reducing the use of traditional insecticides (3–5). Additional research will be needed to evaluate apple pest suppression using hail netting for a range of geographies, fruit crops, marketing strategies (including organic certification), and insurance schemes to further document economic tradeoffs and to quantify potential environmental benefits.

## Data availability statement

The raw data supporting the conclusions of this article will be made available by the authors, without undue reservation.

## Author contributions

GD: Conceptualization, Data curation, Formal Analysis, Funding acquisition, Investigation, Methodology, Project administration, Writing – original draft, Writing – review & editing. SN: Formal Analysis, Methodology, Writing – review & editing. JJ: Investigation, Resources, Writing – review & editing. AK: Funding acquisition, Investigation, Project administration, Writing – review & editing. WH: Conceptualization, Funding acquisition, Investigation, Project administration, Writing – review & editing.
